# GLUT1 production in cancer cells: a tragedy of the commons

**DOI:** 10.1038/s41540-022-00229-6

**Published:** 2022-06-29

**Authors:** Anuraag Bukkuri, Robert A. Gatenby, Joel S. Brown

**Affiliations:** 1 Cancer Biology and Evolution Program and Department of Integrated Mathematical Oncology, Moffitt Cancer Center, Tampa, FL USA; 2 Department of Radiology, Moffitt Cancer Center, Tampa, FL USA

**Keywords:** Cancer, Cancer

## Abstract

The tragedy of the commons occurs when competition among individual members of a group leads to overexploitation of a shared resource to the detriment of the overall population. We hypothesize that cancer cells may engage in a tragedy of the commons when competing for a shared resource such as glucose. To formalize this notion, we create a game theoretic model of glucose uptake based on a cell’s investment in transporters relative to that of its neighboring cells. We show that production of transporters per cell increases as the number of competing cells in a microenvironment increases and nutrient uptake per cell decreases. Furthermore, the greater the resource availability, the more intense the tragedy of the commons at the ESS. Based on our simulations, cancer cells produce 2.2–2.7 times more glucose transporters than would produce optimal fitness for all group members. A tragedy of the commons affords novel therapeutic strategies. By simulating GLUT1 inhibitor and glucose deprivation treatments, we demonstrate a synergistic combination with standard-of-care therapies, while also displaying the existence of a trade-off between competition among cancer cells and depression of their gain function. Assuming cancer cell transporter production is heritable, we then show the potential for a sucker’s gambit therapy by exploiting this trade-off. By strategically changing environmental conditions, we can take advantage of cellular competition and gain function depression.

## Introduction

Glucose is a primary energy source for mammalian cells, serving as fuel for creating ATP through oxidative phosphorylation and glycolysis and as a carbon source for amino acids, nucleotides, and lipids^1,2^. Cells acquire glucose through facilitated diffusion and can express several membrane transporters that promote uptake of glucose and other sugars^3,4^. Widespread clinical application of FdG-PET imaging has demonstrated that most cancer cells consume glucose at a rate that is more than twice their normal counterparts. Immunohistochemical studies demonstrate that cancer cells typically have increased expression of glucose membrane transporters such as GLUT1, which correlates with rapid proliferation and metastatic potential^5^. Furthermore, many cancer cells exhibit the “Warburg phenomenon” in which they metabolize glucose anaerobically even in the presence of oxygen. The reduced efficiency of aerobic glycolysis (2 moles ATP/mol glucose) compared to oxidative phosphorylation (30 moles ATP/mol glucose) (termed “aerobic glycolysis”) requires increased glucose flux for adequate ATP production^6–8^.

Glucose is but one of numerous resources available to cancer cells. In addition to essential amino acids and trace nutrients, other resources (e.g., amino acids, fatty acids, and acetate) provide carbon sources to fuel biosynthesis, growth, and proliferation^9,10^. Glucose can act as either a substitutable, complementary, or hemi-essential resource with other resources^11^. As a consequence, glucose is likely a limiting or co-limiting resource, and cancer cells are known to greatly increase their uptake of glucose relative to normal cells^12^. In vitro, cancer cells increase their proliferation rates with increased glucose availability. Accordingly, glucose oversupply has been associated with higher cancer risk^13^, and researchers have found that high blood glucose levels are associated with increased risk and poor prognosis in prostate^14^, gallbladder^15^, colorectal^16^, and pancreatic cancer^17^. Here, we assume that glucose is a limiting resource.

At first glance, a cancer cells’ investment into transporters for glucose uptake is a straightforward adaptation to compensate for the inefficient production of energy by aerobic glycolysis and to meet metabolic demands. However, glucose represents a “public good” or shared resource supplied by blood perfusion. Furthermore, cancers typically generate poorly organized vascular networks resulting in chaotic blood flow with reduced, temporally fluctuating delivery of nutrients. When each cancer cell is competing with its neighbors for a limited supply of glucose, individual investment in uptake is subject to evolutionary cost/benefit dynamics. For example, a cancer cell might consume more glucose than necessary to meet its metabolic demands simply to reduce the glucose supply for and general fitness of competing cancer cells in its microenvironment. Here, the cost of increased glucose transport is weighed against the benefit of reducing fitness of the cell’s competitors.

These dynamics result in conflict between the best outcome for the group and the optimal outcome for competing individuals within the group. The former is termed the team-optimum strategy, in which selection acts at the group level, providing a strategy that maximizes the fitness of the entire population. That is, the team-optimum strategy calls for maximally efficient nutrient utilization so that the limited supply of glucose maintains the highest possible fitness for each member. However, in cancer, the individual cell is the unit of natural selection and each cell’s proliferation is dependent on the difference between its fitness and that of its competitors. Thus, evolutionary strategies include increases in expression of GLUT1 and other transporters. Although the cost of this increase in glucose transport may reduce the fitness of an individual, the benefit accrued through “stealing” glucose from its neighbors to decrease their fitness may result in a net evolutionary benefit. To further understand this difference, consider an example of cows grazing on a field. The team-optimal strategy describes the amount of grass that each cow should eat to maximize the growth of the entire cow population. However, selection acts on the individual cow, so it is evolutionarily favorable for each cow to consume more grass than it would under the team-optimum strategy even if it provides only marginal benefits, as this improves its relative fitness within the population.

Although such a strategy may be initially successful, it inevitably leads to a reciprocal, escalating response from neighbors. This “arms race” among individuals, which consumes resources to synthesize and maintain membrane glucose transporters, ultimately leads to lower individual fitness than what would be achievable as a team-optimum: a tragedy of the commons^18–20^. Nature abounds with examples of the tragedy of the commons, from competing parasites that selfishly destroy their mutual host^21^ to sexual conflict, in which males may greatly harm females when competing with other males for mating^22^. These general “prisoner’s dilemma” game theoretic dynamics have been applied to explain cancer cells’ metabolic transition from a Pasteur to Warburg effect and from a Warburg effect to intraspecific competition^6,23,24^. Indeed, cancer itself can be viewed as a tragedy of the commons: cancer develops from an initially normal mass of cells, all working cooperatively, controlling their own rates of cell growth and division. However, once these cells become cancerous, they act selfishly, hoarding more nutrients and growth factors than needed for efficient resource consumption, to the detriment of all cells in the microenvironment^25^.

We hypothesize these dynamics play an important role in cancer biology. In evolutionary game theory, the primary equilibrium concept is the evolutionarily stable strategy (ESS) that describes the expected outcome of evolution by natural selection (see ref. ^26^ for nuances on this point). Once adopted by most individuals in a population, the ESS cannot be invaded by any rare alternative strategy. Specifically, at the ESS, no cancer cell can increase its fitness by unilaterally changing its own strategy^27,28^. For more details on equilibrium concepts and a more thorough introduction to evolutionary game theory, we refer interested readers to ref. ^29^. As noted above, when a common good such as glucose is available to a group of cancer cells, there is a team-optimum strategy which results in the highest collective fitness to the entire population. However, if cancer cells individually compete for glucose and other nutrients, a tragedy of the commons will result in an overinvestment in glucose transporters and a lower individual fitness. Recognizing this vulnerability may permit treatment strategies to exploit cell-cell competition and administer treatments to steer cancer cell glucose transporter expression away from a team-optimum and to levels even more extreme than those at the ESS. Cancer cells engaging in a tragedy of the commons, by evolving a less fit phenotype, can favor the patient and inducing or exacerbating such tragedies offers novel therapeutic strategies.

To investigate the role of tragedy of the commons in cancer, we create a game theoretic model, informed by estimates of glucose membrane transporter (termed “GLUT1”) counts, of glucose uptake and utilization in groups of cancer cells in which glucose is a public good necessary for optimal fitness. We focus on the role of GLUT1 in individual cells as they compete for a limited supply of glucose. We compare the ESS that emerges from these evolutionary competitions with the team-optimum to determine the presence of the tragedy of the commons and its fitness cost. We simulate GLUT1 inhibitor and glucose starvation treatments and show the synergistic impact cancer cell competition and neighborhood sizes have on cancer cell fitness and transporter expression. We also show the existence of a trade-off between inducing competition among cancer cells and reduction of their gain function. Assuming cancer cell transporter production is purely hereditary, we show the utility of using sucker’s gambit therapy^30^ to exploit the cancer cell’s proclivity to engage in tragedy of the commons. Such a therapy aims to exploit and change the game^31^ and induces even lower payoffs than those at the ESS.

## Results

### Model analysis

#### Team-optimum versus ESS investment in transporters

Both in the theoretical model we constructed (see Methods) and likely in actual tumors, the upregulation and maintenance of glucose transporters have a game theoretic component. When an individual cell increases its transporters, it benefits in two ways. First, it accesses glucose that otherwise would have gone unharvested by any cells. Second, it accesses glucose that otherwise would have been harvested by other cells, thus denying them resources. It is this second aspect that creates the tragedy of the commons in which the evolutionarily stable strategy (ESS) diverges from the team optimum.

The ESS and the Tragedy of the Commons: To determine the ESS of transporter production, u*, we must find a *v* such that *v* satisfies the ESS maximum principle^29,32^. Specifically, *G* must be maximized with respect to *v* when all members of the population, including the focal individual, are using u*. To do this, we first determine the fitness gradient of *G* with respect to *v* in the model outlined in the Methods section as follows:

1 $$\frac{\partial G}{\partial v}=\frac{Rav{e}^{-av}}{x}+\frac{RN(1-{e}^{-av})(x-v)}{{x}^{2}}-k$$

Then, we set this gradient equal to zero and solve with *v* = u*:

2 $$k=\frac{Ra{e}^{-av}}{N}+\frac{N-1}{N}\frac{R(1-{e}^{-av})}{v}$$

To verify that this equilibrium occurs at a maximum on the fitness landscape, we must confirm that *G* is concave with respect to *v*. To do this, we compute the second derivative of *G* as follows:

3 $$\frac{{\partial }^{2}G}{\partial {v}^{2}}=\frac{R}{x}\left(\frac{2N(v-x)(1-{e}^{-av})}{{x}^{2}}+\frac{2a(x-v){e}^{-av}}{x}-\frac{{a}^{2}v{e}^{-av}}{N}\right)$$

Once again, we let *v* = u* to get:

4 $$\frac{{\partial }^{2}G}{\partial {v}^{2}}=\frac{R}{x}\left(\frac{2(1-{e}^{-av})}{v}\frac{1-N}{N}+\frac{a{e}^{-av}(2(N-1)-av)}{N}\right)$$

Assuming biologically reasonable parameter values, $\frac{{\partial }^{2}G}{\partial {v}^{2}} \,<\, 0$, implying that this equilibrium indeed lies on a peak of the fitness landscape. From Eq. (2), we see that as the number of competing cells in the microenvironment increases, each individual cell weights its production more toward the average return per transporter (second term) rather than the marginal return per transporter (first term). For a negatively accelerating gain function, the average is always greater than the marginal. Thus, transporter production per individual increases with an increase in competing cell counts.

The team-optimum: Now we determine when collective nutrient uptake is maximized. To do this, we must find the u* for which *NG*(*u*, *v*, *N*), is maximized with respect to total transporter production. Since $\frac{\partial NG(u,v,N)}{\partial x}=N\frac{\partial G(u,v,N)}{\partial x}$, we have

5 $$\frac{\partial G}{\partial x}=\frac{Ra{e}^{-av}-k}{N}$$

Thus, we have

6 $$\frac{\partial NG}{\partial x}=Ra{e}^{-av}-k$$

Setting the gradient equal to 0, we have:

7 $$k=Ra{e}^{-av}$$

To confirm that this is the team optimum, we assess whether *NG* is maximized with respect to *x*:

8 $$\frac{{\partial }^{2}NG}{\partial {x}^{2}}=\frac{-R{a}^{2}{e}^{-av}}{N}$$

Thus, since $\frac{{\partial }^{2}NG}{\partial {x}^{2}} \,<\, 0$, we conclude that this equilibrium is indeed the team *optimum*. Note that this is analogous to the ESS transporter production when *N* = 1, in which average benefit vanishes and production of transporters is dependent entirely on marginal benefit. Thus, total nutrient uptake is maximized when *N* = 1. Putting these two pieces together, we see the emergence of a tragedy of the commons situation: in the presence of competing cells, cells ramp up production of transporters but, in the process, also reduce overall nutrient uptake.

#### Model parametrization

It is known that there are 500,000−700,000 GLUT1 transporters on the surface of red blood cells^33^, but analogous data is not available for cancer cells. We estimate this quantity for cancer cells by using the following relationship, derived from the Michaelis–Menten equation:

9 $$[E]=\frac{{V}_{max}}{{K}_{cat}}$$

in which [*E*] is the concentration of the enzyme, *V*_*max*_ is the maximum rate of the reaction, and *K*_*cat*_ is the catalytic constant: the maximum number of chemical conversions of substrate molecules per unit time per catalytic site for a given enzyme concentration. In our case, [*E*] is the concentration of GLUT1 transporters (given in mol/gram of cell) and *K*_*cat*_ represents the number of glucose molecules that can enter the cell through the GLUT1 transporters per unit time.

The *K*_*cat*_ value for GLUT1 for 3-O-methylglucose (3-OMG) is 123*s*^−1^^34,35^. Through analysis of dose-response data, a single functional component with a *V*_*max*_ of 14nmol/10^6^cells/min for 3-OMG was determined for human choroid plexus papilloma (HCPP) cells^36^. We assume here that there are 10^8^ cancer cells in a gram of tumor tissue^37^. From this, we can estimate the concentration of GLUT1 transporters in mol per gram of cancer cell, denoted by [*GLUT*1]_*HCPP*_, as follows:

10 $${[GLUT1]}_{HCPP}=\frac{14\,{{{\rm{nmol}}}}/\min /1{0}^{6}\,{{{\rm{cells}}}}}{7380/\min }\approx 0.002\,{{{\rm{nmol}}}}/1{0}^{6}\,{{{\rm{cells}}}}=0.2\,{{{\rm{nmol}}}}/{{{\rm{g}}}}$$

Note that the higher expression of GLUT1 in the papilloma cell is consistent with medical findings, which suggest higher glucose utilization by tumor cells than normal somatic ones. Now, we can estimate the number of GLUT1 transporters on a cancer cell, denoted by *GLUT*1_*HCPP*_, as follows:

11 $$GLUT{1}_{HCPP}=\frac{0.2\,{{{\rm{nmol}}}}/{{{\rm{g}}}}* {N}_{A}}{1{0}^{8}\,{{{\rm{cells}}}}/{{{\rm{g}}}}}=1,204,428\,{{{\rm{GLUT}}}}1/{{{\rm{cell}}}}$$

Thus, assuming a 50% variance in both directions, we derive the range of GLUT1 transporter count on cancer cells to be 602,214 to 1,806,642. Using these estimates, we parameterize our model as shown in Table 1.

**Table 1:** Parameter values used in numerical simulations.

Parameter	Meaning	Value
*k*	Cost of Transporter Production	0.0055
*R*	Resource Availability	10,000
*a*	Encounter Rate	2e−6

#### Transporter equilibria and payoff simulations

**Fig. 1:**
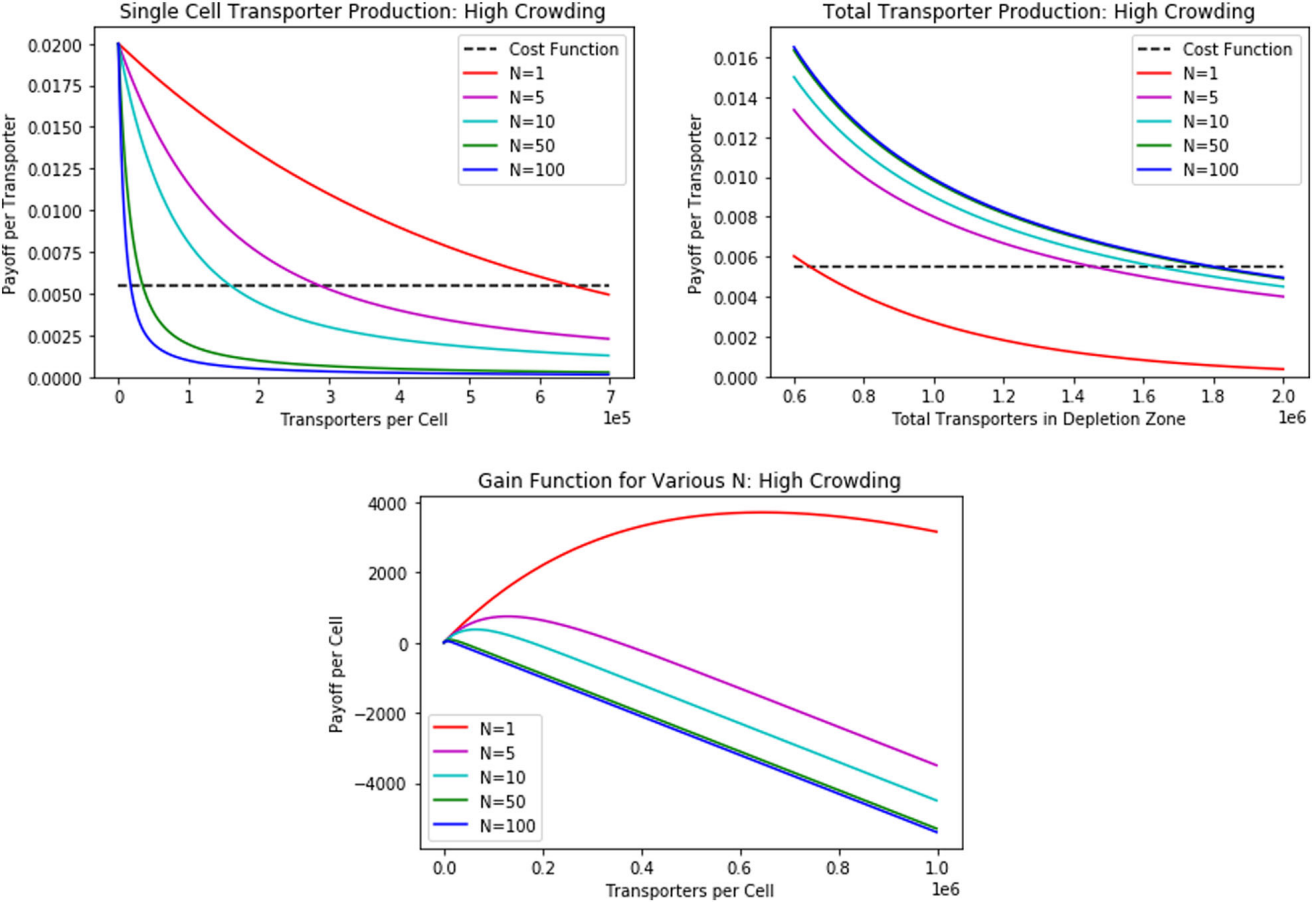
How changing crowding (see Fig. 12) influences transporter equilibria per cell, transporter equilibria per depletion zone, and payoff curve per cell for various *N*. At the team-optimum, the total number of transporters remain constant for various *N* (transporters per cell would adjust accordingly). At the ESS, the total number of transporters in the neighborhood increases while the number of transporters per cell decreases as a function of *N* (though this decrease is much lower than it would be if maintaining the team-optimum). The payoff to each cancer cell depends on both a unique payoff curve (determined by the number of competing cells in the neighborhood) and the location of the cell on this curve (determined by the ESS transporter count).

Using these parameters, we can compare the number of glucose transporters at the ESS with that at the team-optimum. We can evaluate these equilibria under high crowding and varying sizes of the depletion zone. Since we cannot find an explicit solution for the ESS equilibrium and we know that the team-optimum is analogous to the ESS equilibrium for *N* = 1, we simply plot the equilibrium for various values of *N*. First, we consider the high crowding assumption simulations, depicted in Fig. 1.

**Fig. 2:**
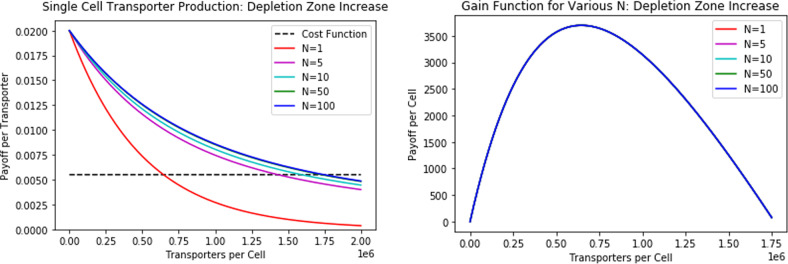
How changing the depletion zone (see Fig. 12) influences transporter equilibria per cell, transporter equilibria per depletion zone, and payoff curve per cell for various numbers of neighbors *N*. At the team-optimum (analogous to the *N* = 1 curves), the total number of transporters remains constant for various *N*. At the ESS, the number of transporters per cell increases as a function of *N*. Since the payoff curve per cell is the same regardless of *N*, the payoff to each cell depends solely on the location of the cell on the curve (determined by the ESS transporter count).

In these plots, the intersection of each curve with the cost function denotes the transporter equilibria. We notice a more subtle, but nonetheless severe, tragedy of the commons. With an increase in competing cells, the total number of transporters summed across all cells increases while the number of transporters per cell decreases. Considering the gain function plot, we note that a unique payoff curve per cell exists for each *N*: the payoff for a cell depends both on the payoff curve of the depletion zone and the equilibrium number of transporters. This determines the exact location of the cancer cell on the curve, thereby providing the payoff. At the team-optimum, the total number of transporters summed across all cells remains equal to the ESS of *N* = 1. Thus, the transporters per cell at the team-optimum declines much more rapidly than that of the ESS as crowding increases. Once *N* > 1, the ESS results in the over-harvesting of nutrients as a consequence of over-producing transporters relative to the team-optimum. Now, we simulate changes in the depletion zone (Fig. 2).

With changes in the size of the depletion zone, the transporter production at the ESS is strictly greater than the team-optimum when at least one competing cell is present. As the size of the depletion zone increases, the team-optimum transporter production per cell remains unchanged while that of the ESS continues to increase. Unlike the crowding assumption, the number of cells in the depletion zone does not influence the payoff curve per cell: the cell’s payoff is solely determined by where on the curve the cell’s transporter equilibrium lies. As the depletion zone and the number of competing cells increase, the payoff at the ESS declines and diverges more and more from the per cell payoff at the team-optimum. Thus, the tragedy of the commons becomes more intense as the depletion zone increases in size. We also note that the change in equilibrium transporter value as a function of *N* seems to follow a roughly logistic trend: equilibrium transporter values for low values of *N* differ much more than for high values of *N*.

To explore this further, we computed the per cell payoff and transporter production at the ESS for a wide range of *N* (Table 2). Note that for a fixed density of cancer cells, the depletion zone is directly proportional to *N*. The team-optimum transporter production and payoff, for all values of *N*, is the same as the ESS transporter equilibrium for *N* = 1.

**Table 2:** Under different levels of crowding, the ESS transporter equilibrium and payoffs for various values of *N*.

*N*	ESS Transporter/Cell Equilibrium	ESS Payoff/Cell
1	645,492	3700
5	1,430,884	1558
10	1,592,629	827
50	1,729,743	172
100	1,747,277	86

**Fig. 3:**
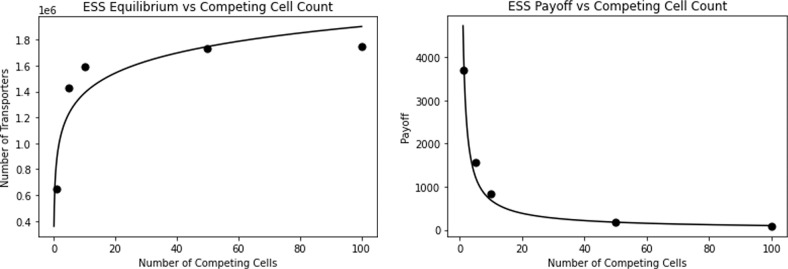
Nutrient uptake and transporter production as functions of competing cells. ESS transporter equilibrium increases in a logistic manner while the ESS payoff per cell deceases in a power-law manner as a function of *N*.

To gain a better understanding of this data, we created plots of the nutrient uptake and ESS transporter equilibria as influenced by the number of competing cells within a focal cell’s depletion zone (Fig. 3). Here, we assume that the increase is due to a larger depletion zone and not through a change in cell density. A large depletion zone exacerbates the tragedy of the commons.

From Fig. 3, we notice that the payoff per cell decreases in a power-law fashion and the transporter equilibria increases in a logistic fashion with an increase in competing cells. Therapeutically, this implies that strategies which promote clustering of cancer cells may be highly effective towards disease eradication: through cell-cell competition, the cancer cells overproduce transporters, driving down their own nutrient uptake of glucose in the process. Cancer cells depend on glucose for aerobic and anaerobic respiration, ATP production, and the construction of functional and structural molecules. Enhancing competition may greatly reduce cell proliferation relative to what it would be at a team-optimum^2,38^. If cancer cells engage in this tragedy of the commons, then exploiting or encouraging this therapeutically could work to the patient’s advantage.

### Modeling therapy

#### GLUT1 inhibitors

**Fig. 4:**
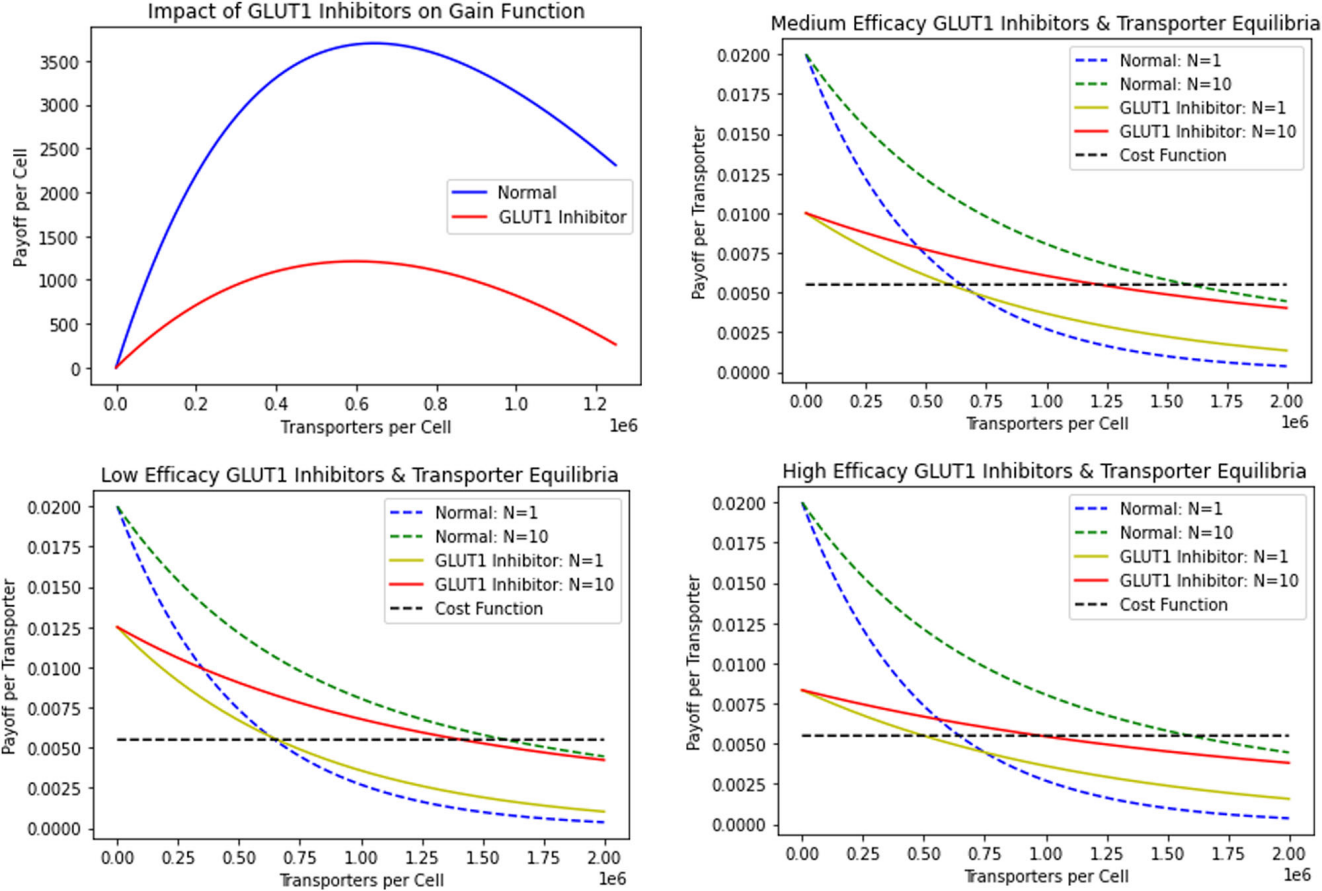
Impact of GLUT1 inhibitor treatment. A lower payoff curve per cell results from treatment. The ESS number of transporters per cell is similar to and lower under GLUT1 treatment than under no treatment for *N* = 1 and *N* = 10, respectively.

Therapies exist that target glucose uptake by cancer cells. One class includes GLUT inhibitors that prevent the GLUT channels on cancer cells from uptaking glucose^1,39^. Mechanistically, this can be done through competitive (STF-31, Genistein, Fasentin) or noncompetitive (Ritonavir, Resveratrol) inhibition^5,40–43^. Both mechanisms cause cells to uptake fewer glucose molecules through their transporters, while the cell still pays the same cost of producing and maintaining them. Medically, these drugs have been shown to result in apoptosis and decreased cancer cell proliferation^44,45^. In our model, this is analogous to decreasing the encounter rate, *a*. Under low (*a* = 1/800, 000 per time), medium (*a* = 1/1, 000, 000 per time), and high (*a* = 1/1, 200, 000 per time) therapeutic efficacy, we simulate the treatment in Fig. 4. In the following simulations, the number of competing cells is set to either 1 or 10, though the same qualitative trends result from other values of *N*.

As we can see in Fig. 4, the payoff curve for cells under GLUT1 inhibitor treatment is strictly lower than that for the untreated cells for any positive number of transporters on the cell. Under all therapeutic efficacies, the number of transporters produced by GLUT1 inhibitor treated cells (per the ESS equilibrium) is similar to the equilibrium for non-treated cells in the *N* = 1 case. In the *N* = 10 case, the transporter count per cells for treated cells is clearly lower than that of non-treated cells. In particular, if we interpret competitiveness as the difference between the ESS and team-optimum equilibria, the cancer cells become less competitive with each other as *a* decreases. The difference between the *N* = 1 (analogous to the team-optimum) and *N* = 10 ESS transporter numbers declines in the GLUT1 inhibitor treated cells as relative to the untreated cells. This difference can also be seen across therapeutic efficacies: the more effective the treatment, the smaller the difference between the ESS and team-optimum is. This means that GLUT1 inhibitors, though effective at decreasing nutrient uptake and the overall payoff to cells, lose therapeutic efficacy because the cancer cells shift closer towards the team optimum as they engage less in the tragedy of the commons. In a sense, this therapy rescues the cancer cells from themselves. To further investigate the impact of treatment on transporter equilibria and payoffs, we compute the ESS equilibrium and payoff of treated cells under medium efficacy of GLUT1 inhibitors for various values of the neighborhood size *N* (Table 3).

**Table 3:** GLUT1 inhibitor ESS transporter equilibria and payoffs.

*N*	ESS Transporter Equilibrium/Cell	ESS Payoff/Cell
1	597,837	1212
5	1,106,639	607
10	1,217,628	344
50	1,317,444	76
100	1,330,640	38

**Fig. 5:**
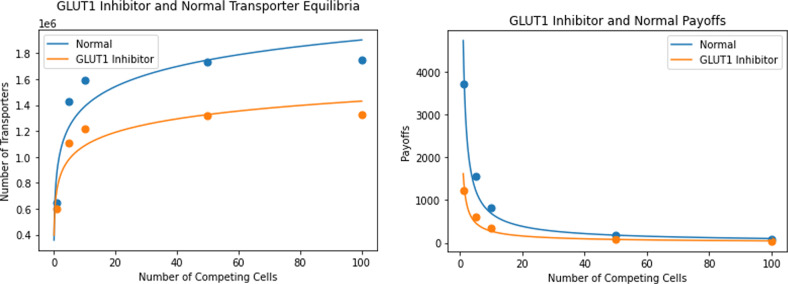
GLUT1 inhibitors vs normal transporter equilibria and payoffs. The left panel plots the number of transporters at ESS as a function of the number of neighboring cells. The right panel plots ESS payoff as a function of neighboring cells. Cancer cells under GLUT1 inhibitor treatment produce fewer transporters and have a lower payoff than untreated cancer cells.

The team-optimum and payoff, for all values of *N*, is analogous to the ESS case for *N* = 1. As *N* increases, the divergence between the payoffs to the team-optimum and ESS increases. Note that the tragedy of the commons increases with the number of competing cells. Even as payoffs decline, each produces even more transporters at its ESS as *N* increases. Clustering of cancer cells overall boosts the efficacy of the GLUT1 inhibitor treatment (Fig. 5).

From Fig. 5, we see that, for all *N*, cancer cells treated with the GLUT1 inhibitor produce fewer transporters and have lower payoffs than untreated cancer cells. More importantly, the reduction in transporter production caused by the treatment reduces the extent to which cancer cells engage in a tragedy of the commons. As seen by the slopes of the curves, as nutrient encounter rate decreases from treatment, the cancer cells become less competitive with each other. The GLUT1 inhibitor directly reduces tumor size by making cancer cells less effective foragers. However, this direct effect is mitigated as the cancer cells’ new ESS involves less of a tragedy of the commons. Thus, clustering of cancer cells helps drive an overproduction of transporters and reduction in payoff at the ESS. This effect is less pronounced for treated than for untreated cells, reducing the overall efficacy of the treatment.

#### Glucose starvation

**Fig. 6:**
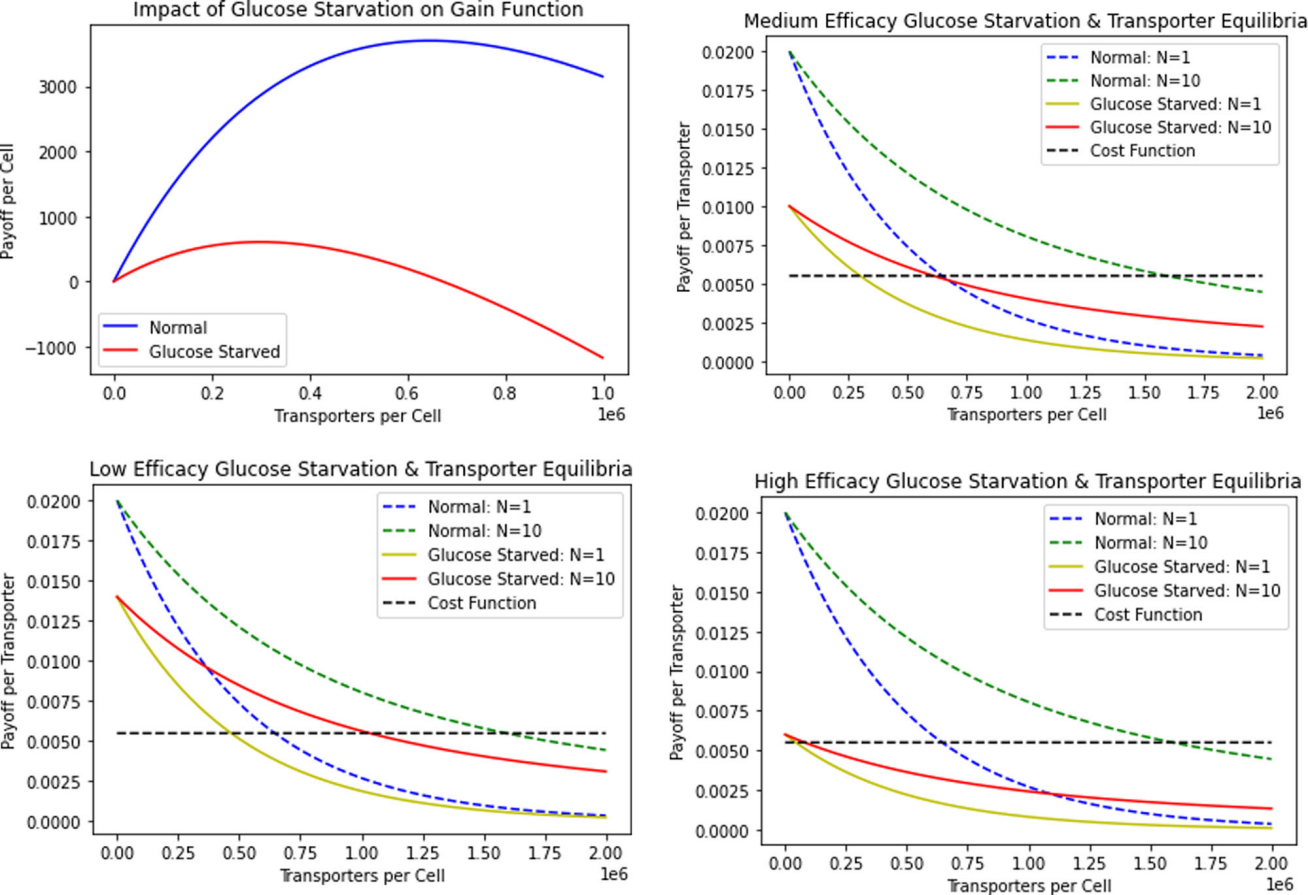
Impact of glucose starvation. Therapy results in a lower payoff curve per cell in a glucose-starved environment. The ESS number of transporters per cell is lower under glucose starvation than under normal conditions.

Glucose starvation provides another approach to cancer treatment, such as with the use of 2-deoxy-D-glucose to enhance cancer therapies^46,47^. Because cancer cells uptake and rely on glucose to a much greater extent than normal cells, glucose starvation preferentially results in cytotoxicity through oxidative stress mechanisms in cancer cells relative to normal ones^48,49^. Cancer cells may experience chronic oxidative stress relative to normal cells. In response, cancer cells upregulate glucose metabolism to produce more pyruvate and NADPH. This protects against hydroperoxide-induced toxicities^49^. Thus, glucose deprivation is expected to inhibit the pathways that cancer cells use to protect against higher steady-state levels of hydroperoxides. In our model, this treatment can be modeled by reducing the amount of available resources, *R*. We simulate low (*R* = 7000), medium (*R* = 5000), and high (*R* = 3000) therapeutic efficacy of the glucose starvation treatment in Fig. 6.

We see similar trends in Fig. 6 as we did in Fig. 4. We see an even more pronounced payoff curve depression in the cells under glucose starvation treatment, which selects for greatly reduced transporter numbers for both the *N* = 1 and *N* = 10 cases across all therapeutic efficacies. Furthermore, as above, notice that as *R* is decreased, cancer cells seem to become much less competitive with each other, meaning that glucose starvation, while effective as a treatment, is buffered by this reduced competition. As will be shown later, this trend of a trade-off between a depression of the payoff curve and a decrease in competitiveness among cancer cells is a general one. It is important to recognize, as evidenced by Figs. 4 and 6, that a change in resource availability has a larger impact on the transporter equilibria and payoff curve per cell than a change in nutrient encounter rate. To further investigate the impact of treatment on transporter equilibria and payoffs, we compute the ESS number of transporters and payoff of treated cells under medium efficacy of glucose starvation for various values of the neighborhood size *N* (Table 4).

**Table 4:** Glucose starvation ESS transporter equilibria and payoffs.

*N*	ESS transporter/cell equilibrium	ESS Payoff/Cell
1	298,919	606
5	553,319	303
10	608,814	172
50	658,722	38
100	665,320	19

**Fig. 7:**
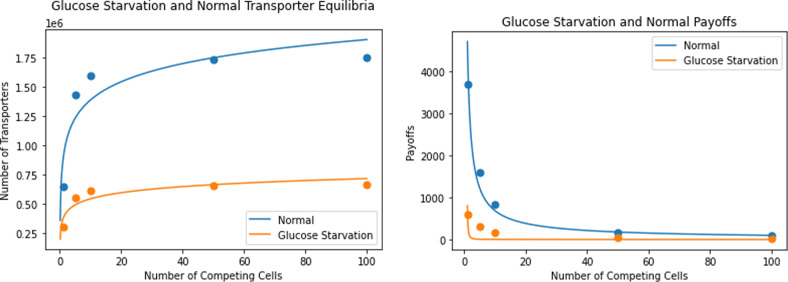
Glucose starvation vs normal transporter equilibria and payoffs. The left panel plots the number of transporters at ESS as a function of the number of neighboring cells. The right panel plots ESS payoff as a function of neighboring cells. Glucose-starved cancer cells produce fewer transporters and have a lower payoff than cancer cells in normal conditions.

The team-optimum and payoff, for all *N*, equals the ESS for *N* = 1. As *N* increases, the difference in payoffs between the ESS and team-optimum also increases. Again, the tragedy of the commons increases with the number of neighboring cancer cells. Even as payoffs decrease, each cell produces more transporters at its ESS as *N* increases. Cancer cell clustering increases the efficacy of the glucose starvation treatment (Fig. 7).

From Fig. 7, we see that glucose-starved cancer cells produce fewer transporters and have lower payoffs than untreated cells for all *N*. Like the GLUT1 inhibitor case, when resource availability is reduced, cancer cells become less competitive with one another, detracting from the efficacy of cell clustering in the treatment. Experimentally, studies measuring how GLUT expression changes in response to glucose availability have been inconclusive. For example, one study on membrane GLUT expression in thyroid cancer cells in hypo-, normo-, and hyperglycemic conditions found that, in FTC-133 cells, plasma membrane GLUT1 expression increased with increasing glucose concentration, while in 8305c cells, it decreased^50^. In a study on diabetic retinopathy, both total and membrane GLUT1 expression increased in response to hyperglycemic conditions^51^. Another study investigating expression patterns of glucose transporters in the rat eye lens found no detectable change in GLUT1 transcript levels in response to hyperglycemia, though an upregulation of GLUT3 (a higher affinity glucose transporter found in cortical fiber cells) was noticed^52^. Reasons for these discrepancies have not been determined, suggesting that further experimental work must be performed to more clearly understand the conditions and cell types under which these trends in plasma membrane GLUT expression occur. Briefly, cancer cells could be grown in normoglycemic conditions (5 mM glucose) for 48 h. These cells could then be passaged into hypoglycemic (2 mM), normoglycemic (5 mM), and hyperglycemic (25 mM) conditions. To assess expression plasticity, GLUT1 expression levels could be monitored over time with Western blotting. The impact of depletion zone scaling assumptions could be explored through the use of a matrigel cell culture matrix vs. standard aqueous media. From the model, we would expect to see an eventual increase in GLUT1 expression with hyperglycemic conditions.

#### Sucker’s gambit

**Fig. 8:**
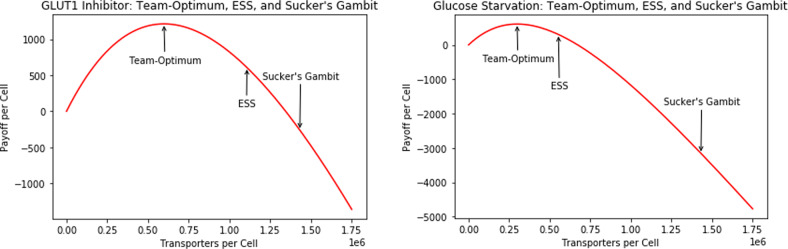
Team-optimum, ESS, and sucker’s gambit equilibria for GLUT1 inhibitor (left) and glucose starvation (right) treatments: *N* = 5 case. The payoff per cell is plotted as a function of the transporters per cell. Under the sucker’s gambit strategy, cancer cells have a lower payoff curve per cell and produce more transporters than both the team-optimum and ESS cases.

When the cancer cells engage in a tragedy of the commons, treatment efficacy becomes reduced as the reduction in cancer cell numbers or resource availability render the tragedy less strong and the cancer cells respond to less competition from neighbors. Is there any way to counteract the trade-off between payoff curve depression and reduced cancer cell competition? One way is with a sucker’s gambit approach^53^. In the context of cancer, a sucker’s gambit refers to the process of changing selection pressures in an evolving tumor to select for phenotypes that are easier to treat^54,55^. This suggests that we may maximize therapeutic impact by first selecting for competitive cancer cell phenotypes and then administering glucose starvation or GLUT1 inhibitor treatments. Consider the situation in which, for example, the concentration of glucose in a depletion zone is increased. Once cancer cells have evolved a higher competitiveness (as is predicted with an increase in resource availability), treatment is administered. We simulate the effects of this strategy on ESS transporter number and payoff under GLUT1 inhibitor and glucose starvation therapy for *N* = 5 in Fig. 8.

From Fig. 8, we see that the cancer cells not only have a depressed gain function, due to a lack of resources, but also produce *many* more transporters than the team-optimum, or even ESS, due to the added competitiveness. As expected, this results in the cancer cells obtaining a much lower, and even negative, payoff.

To implement a successful evolutionary double-bind by alternating therapy will require knowledge of how quickly the cancer cells manifest changes in transporter numbers^29^. The goal is to exploit the rate of change to create glucose rich conditions when transporter numbers are low, and then induce resource poor conditions (through targeted therapy or glucose starvation) just as the cancer cells have switched to high transporter numbers. If transporter production is highly plastic, meaning cells can modify their production of transporters within their lifetime to environmental changes (plasticity hypothesis), this strategy would require rapid therapeutic switching which may be unrealistic. However, if cancer cell transporter production is heritable (requires genetic or epigenetic changes) and only changes over generations of cancer cells, then we would be able to initially induce high levels of competitiveness in glucose-starved environments, before cancer cells evolve to new transporter equilibria.

We now more precisely analyze the efficacy of this strategy in the GLUT1 inhibitor and glucose starvation treatments. Under the plasticity hypothesis, we assume that transporter production immediately adjusts to environmental change. Thus, transporter production and payoffs under this case would be analogous to those under just GLUT1 inhibitor or glucose starvation treatment. To measure efficacy, we can then simply compare equilibria and payoffs under the genetic hypothesis to our controls: the isolated GLUT1 inhibitor and glucose starvation treatments.

**Fig. 9:**
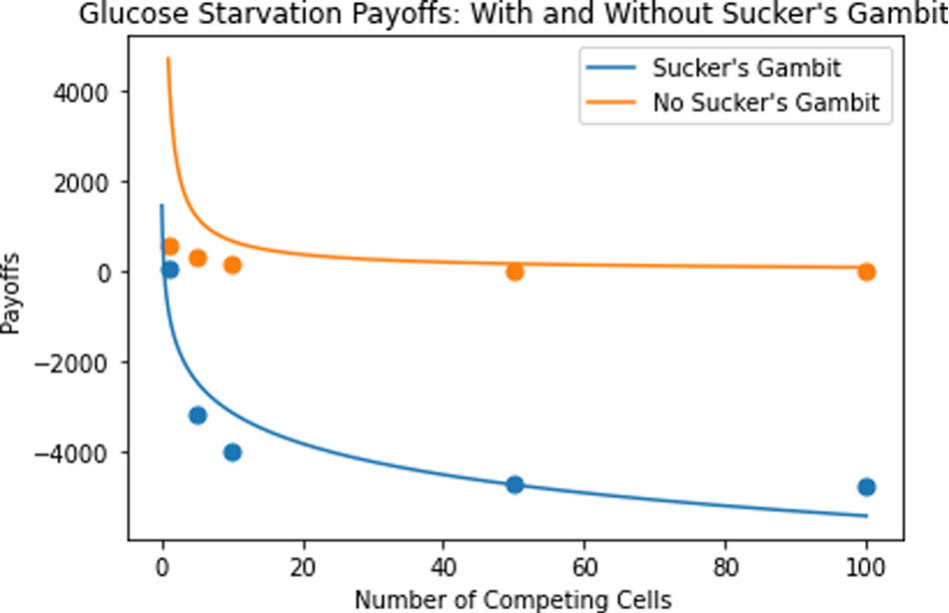
Payoff from the glucose starvation treatment with and without a sucker’s gambit. Payoff is plotted as a function of the number of competing cells. Under the sucker's gambit strategy, we see a clear depression of the payoff curve per cell, with negative payoff values.

We assume cancer cells are maintained at the original resource availability (*R* = 10,000) before inducing glucose starvation, bringing *R* down to 5000. Table 5 represents the ESS equilibria and payoffs under the sucker’s gambit strategy (under the genetic hypothesis) and Fig. 9 compares payoffs for the treatment with and without the gambit. Note that the following trends could be further exaggerated by inducing hyperglycemia through the use of *β*-blockers or protease inhibitors, for instance, before using the glucose starvation treatment^56^.

**Table 5:** ESS transporter equilibria and payoffs under sucker’s gambit strategy: resource availability modification.

*N*	ESS Transporter/Cell Equilibrium	ESS Payoff/Cell
1	645,492	75
5	1,430,884	−3156
10	1,592,629	−3966
50	1,729,743	−4671
100	1,747,277	−4762

Under the sucker’s gambit, we were able to take advantage of both cancer cell competition, as evidenced by the increased ESS transporter numbers per cell with an increase in *N*, and the decreased payoff per cell associated with glucose starvation treatment. The result is striking: negative payoffs were detected for all values of *N* tested above 1. Maintaining negative payoffs could result in cure.

**Fig. 10:**
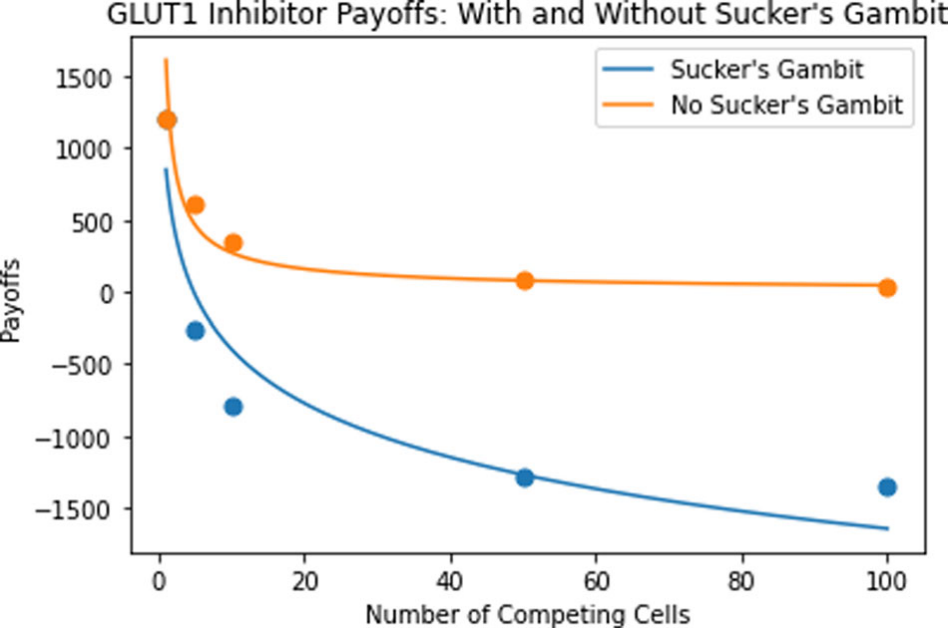
GLUT1 inhibitor treatment payoffs with and without sucker’s gambit. Payoffs per cell are plotted as a function of number of competing cells. Under the sucker's gambit strategy, we see a clear depression of the payoff curve per cell, with negative payoff values.

Similarly, assume cancer cells are maintained at the original nutrient encounter rate, before a GLUT1 inhibitor treatment is administered. Assuming a treatment efficacy of 50%, Table 6 represents the ESS equilibria and payoffs for such a strategy for cells under the genetic hypothesis and Fig. 10 compares treatment payoffs with and without sucker’s gambit incorporation. Again, note that these trends could be further exaggerated by first activating GLUT1 through the use of osmotic or metabolic stimuli such as M*β*CD or azide, before administering the GLUT1 inhibitors^57,58^.

**Table 6:** ESS transporter equilibria and payoffs under sucker’s gambit strategy: nutrient encounter rate modification.

*N*	ESS Transporter/Cell Equilibrium	ESS Payoff/Cell
1	645,492	1206
5	1,430,884	−260
10	1,592,629	−793
50	1,729,743	−1287
100	1,747,277	−1353

Though these results are not as extreme as those for the glucose starvation treatment, we still took advantage of both the competition among cancer cells and the reduced payoff curve of GLUT1 inhibitor treatments. Again, we have negative payoffs for all values of *N* above 1. Thus, when the genetic hypothesis is true, exploiting cancer cell competition through the sucker’s gambit may prove to be an extremely effective therapeutic option for GLUT1 inhibitor and glucose starvation treatments. It is important to remember here that, even under the genetic hypothesis, cancer cells will eventually evolve to new transporter equilibria; hence, it may be required to iterate the sucker’s gambit strategy, alternating between high and low resource availability or nutrient encounter rates for maximal therapeutic impact and eventual cure or maintenance as a chronic disease as envisioned by adaptive therapies^59,60^.

In addition to the transporter equilibria and payoff changes we have analyzed here, we speculate that an overproduction of transporters may have an additional benefit. Drawing from life history theory, there exist trade-offs between somatic and reproductive effort, which arise from competitive allocation of limited resources and energy between different life history traits^61,62^. Thus, cells that are forced to invest much energy and resources (as evidenced by the negative payoffs) in maintenance and growth have less energy to invest in cell division, reducing the growth rate of the tumor overall. Having the cancer cells engage in a tragedy of the commons does not render the cancer benign, but it does render the cancer cells less proliferative than they otherwise could be under a team optimum. This works to the patient’s advantage by slowing tumor growth below its intrinsic maximum.

## Discussion

In this paper, we developed a game theoretical model of cancer cell competition for limited nutrients in the tumor microenvironment. We parametrized our model in the context of glucose uptake by GLUT1 transporters. The model, concepts, and framework, however, are more general. A tragedy of the commons could result from cancer cells competing for other limiting or co-limiting resources that involve upregulating transporters and other uptake machinery for enhancing nutrient uptake^63–66^. Through mathematical analysis, we determined an implicit ESS and an explicit team-optimum and proved the emergence of a tragedy of the commons. We made estimates of the number of GLUT1 transporters on the surface of cancer cells and parameterized our model to fit these estimates. Our simulations confirmed that competition for limited resources among individual cancer cells produce a GLUT1 “arms race” with individual cells increasing glucose uptake in excess of their metabolic demands to reduce nutrient availability for competitors. A decreasing power law relation was found between gain function payoff and competing cell number, while a logistic relation was found between transporter production and competing cell counts. Our simulations show that a tragedy of the commons will consistently emerge in cancer populations resulting in a lower fitness compared to an optimal state in which glucose metabolism is maximally efficient to optimize resource availability for all members of the population. We demonstrate that GLUT1 inhibition and glucose starvation have synergistic impacts on cancer cell fitness through lower payoffs and higher transporter production. A trade-off between cancer cell competition and payoff curve depression was recognized and the efficacy of a sucker’s gambit strategy to circumvent this issue was shown.

Importantly, a tragedy of the commons reduces the fitness of cancer cells and, ironically, the least competitive cells in the competition for public goods are actually the most dangerous to the patient. Note that, unlike traditional public goods games in which competition reduces (but does not disappear) as the number of competitors increases, our tragedy of the commons is exacerbated as the density of competitors in a cancer cell’s neighborhood increases. With this knowledge, researchers and clinicians may focus on creating and administering treatments that increase cancer cell competition, forcing cancer cells to lower their own fitness, in addition to improving current, standard-of-care therapies by inducing higher rates of cancer cell clustering.

Finally, we note that the evolutionary dynamics that produce a tragedy of the commons may occur with other public goods that are used by or produced by cancer cells. For example, the production of angiogenic promoters such as VEGF is subject to cheating dynamics in which cells do not assume the cost of VEGF production but still benefit from production of VEGF by their neighbors. This work will benefit from future experimental work to quantify limitations and costs of cancer cells’ transporter production, the effect of transporter production on cell proliferation rates, synergistic combination of cancer cell glucose transport with standard cancer treatments, and application of the sucker’s gambit strategy for glucose uptake in cancer treatment.

## Methods

### Model creation

Consider a tumor microenvironment in which a finite number of cancer cells obtain nutrients from a finite pool of resources. Uptake of nutrients by one cell decreases the amount available for other cells. The cells uptake these nutrients through transporters on their cell membrane; however, each transporter costs the cell a fixed amount of energy to produce and maintain. Furthermore, due to the nature of uptake kinetics, nutrient uptake increases at a decelerating rate with the number of transporters. As the cell produces more transporters, the marginal benefit of each transporter declines. The questions we seek to answer are the following: given this framework, how many transporters should a cell produce, what are the corresponding payoffs, and how do these compare to what is best for the group (the collective payoff)?

To answer these questions, we draw inspiration from nutrient foraging by plants. They adjust the proliferation, structure, and physiology of their root systems in response to resource availability and the presence of competitors’ roots^67–69^. The root systems of neighboring plants influence the nutrient uptake, and thus fitness, of a focal plant^17,70^. A study performed by ref. ^71^ demonstrated the existence of an intraspecific root proliferation game in the soybean plants *Glycine max*: these plants reduced root production when their roots were the entirety of the competitive environment and exaggerated root production in the presence of other plants’ roots. In cancer, we hypothesize that a similar tragedy of the commons scenario occurs in the context of cellular nutrient uptake and transporter production. To examine this hypothesis, we formalize a cell’s nutrient uptake rate as a function of its transporters and the number of transporters of its competitors using a modified version of the game theoretical model presented in ref. ^71^. We use a fitness generating function approach (*G* function, as in ref. ^29^) to describe the expected success of an individual cancer cell as influenced by its own transporter production and that of others:

12 $$G(v,\hat{u},N)=\frac{v}{x}\phi (x)-C(v)$$

*G* captures cancer cell fitness as the difference between nutrient uptake (first term) and cost of transporter production and maintenance (second term). In this function, *v* is the transporter count of the focal individual, $\hat{u}$ is a vector describing the number of transporters for each of the other cells in the group, *x* = *v* + ∑*u*_*i*_ is the total number of transporters in the group, and *N* is the total number of interacting individuals within the local microenvironment. We use the functional forms *ϕ*(*x*) = *R*(1 − *e*^−*ax*^) and *C*(*v*) = *kv* for nutrient uptake and transporter cost, respectively, where *R* is glucose availability in the tumor microenvironment, *a* measures the encounter probability of a single membrane transporter with a given glucose molecule, and *k* is the cost of producing and maintaining a transporter. In this way, the fitness generating function is both frequency dependent (i.e., individual fitness depends on the trait values of cells in the population: *v*, $\hat{u}$) and density dependent (i.e., individual fitness depends on the number of cells in the microenvironment: *N*). As we discuss in the next section, resource availability (*R*), encounter rate (*a*), and the population size within the microenvironment (*N*) are scale-dependent and influenced by the area encompassed by a cancer cell’s microenvironment.

The bounded exponential form we use for uptake kinetics is appropriate under the assumption that at any given time, each transporter has the same probability as another at encountering any given glucose molecule. This assumes a well mixed system within the neighborhood of cancer cells, and is consistent with prior studies which show that nutrient uptake follows Michaelis–Menten kinetics^72,73^. As the collective number of transporters of the neighborhood of cells goes from zero to infinite, the rate of nutrient harvest goes from zero to *R*, with diminishing returns to each additional transporter.

It is important to make a distinction between *N* and the total number of cancer cells in the tumor. Just as a tree in a large forest only competes with its immediate neighbors and not all of the trees, competition for nutrients among cancer cells only occurs within a small neighborhood of a given cell^74^. We define competition here as a cell’s investment into producing glucose transporters to preempt its neighbors’ uptake. The size of a cancer cell’s neighborhood is determined by the cell’s depletion zone: the area over which a cell can effectively access and deplete nutrients. We let *N* be the number of cells within this zone. For simplicity, we assume that the spatial distribution of cells within the depletion zone is negligible, i.e., all cells in the depletion zone have equal access to nutrients. The collective uptake of nutrients is determined by the collective number of transporters present among the cells of the neighborhood, *x*. An individual cell’s share of this collective harvest is determined by its proportional share of transporters which is *v*/*x*. The second term then gives the cost of investment into the production and maintenance of a cell’s transporters: *kv* in the case of the focal cell.

**Fig. 11:**
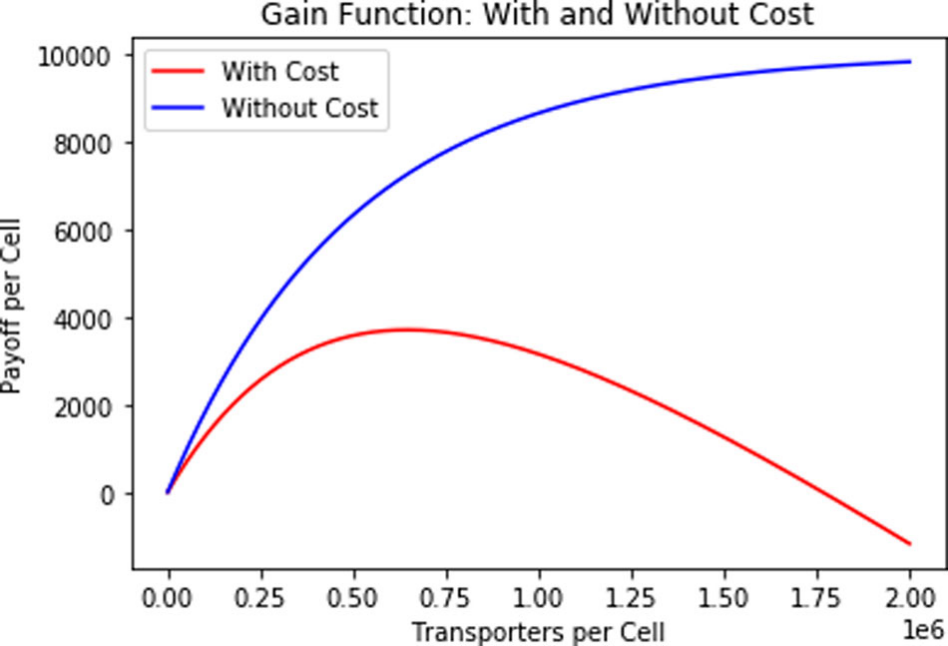
Plots of nutrient uptake. Incorporation of the cost of transporters into the payoff per cell changes the payoff curve from one which is strictly increasing to one with a clear maximum.

The assumed relationship between nutrient uptake and number of transporters, with and without including the cost of producing transporters, is shown in Fig. 11.

### Scaling assumptions

**Fig. 12:**
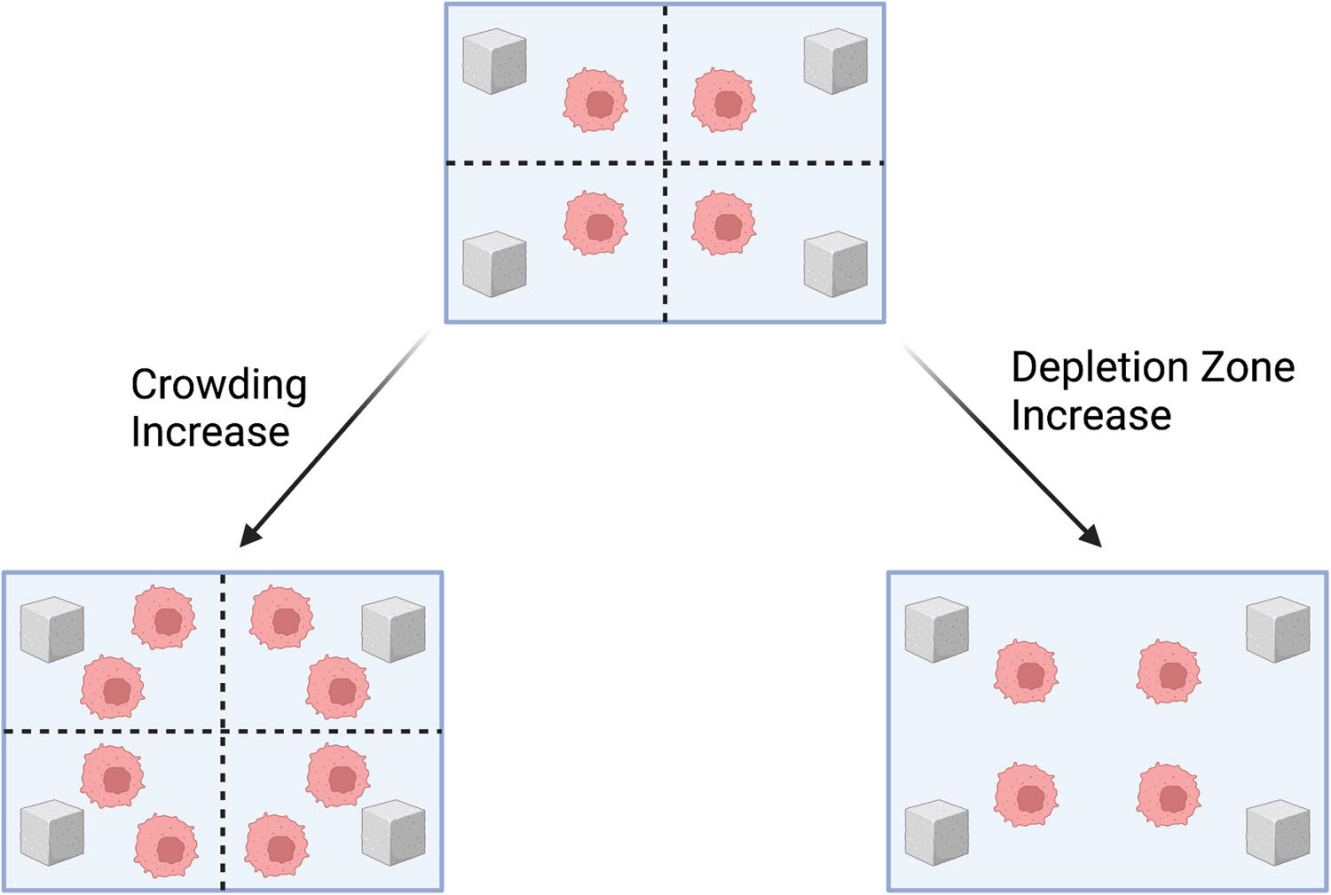
Illustration of the differences between crowding and depletion zone increase assumptions. Sugar cubes represent glucose molecules, the cell depicted is a cancerous one, and the black lines divide the environment into depletion zones. With the “crowding increase" assumption, the size of the depletion zone and nutrient availability are held constant while the cell density per unit area increases, reducing nutrient availability per cell. In the “depletion zone increase" assumption, both nutrient availability and size of the depletion zone are increased, keeping cell density per unit area and nutrient availability per cell constant. Created with BioRender.com.

Before proceeding, we must enforce a scaling rule that tells us how resource availability (*R*) and nutrient encounter rate (*a*) change as the number of cells in a depletion zone (*N*) change. First, with crowding (*crowding increase assumption*), the size of the depletion zone remains fixed and changes in *N* reflect fewer or greater individual cells within this fixed space. As a result, the resource availability, *R*, and nutrient encounter rate of a cell, *a*, remain constant. Second, changes in *N* may reflect changes in the size of the depletion zone (*depletion zone increase assumption*) while the density of cells stays constant. In this case, both *R* and *a* will change in opposite directions. A larger depletion zone results in a higher nutrient availability, but it also decreases the chance of a cell encountering them. These scenarios are depicted below in Fig. 12. Drawing on plant biology, we choose to enforce the latter assumption^71^. Specifically, we assume that *R* is directly proportional to and *a* is inversely proportional to the number of cells in the depletion zone. Under the depletion zone assumption, *R*(*N*) = *RN* and *a*(*N*) = *a*/*N*. With this formulation, notice that resources per cell remain constant: *Nϕ*(*u*) = *ϕ*(*x*).

## Data Availability

Data sharing not applicable to this article as no datasets were generated and analysed during the current study.

## Code availability

Codes used to produce all plots in this paper can be found at the following publicly accessible GitHub page: https://github.com/abukkuri/TOC.
